# Comparative Multi-Omics Analysis and Antitumor Activity of *Phylloporia crataegi* and *Phylloporia fontanesiae*

**DOI:** 10.4014/jmb.2504.04029

**Published:** 2025-08-28

**Authors:** Yan Fu, Yuyue Zhang, Zhihao Guo, Guoli Zhang, Yinghao Zhang, Xuemei Tian

**Affiliations:** Shandong Province Key Laboratory of Applied Mycology, College of Life Sciences, Qingdao Agricultural University, Qingdao 266109, P.R. China

**Keywords:** *Phylloporia crataegi*, *Phylloporia fontanesiae*, antitumor, multi-omics analysis

## Abstract

Wood-decay fungi, particularly those within the *Phylloporia* genus, have attracted considerable interest due to their diverse bioactivities, including antitumor, immune-modulatory, anti-inflammatory, antiviral, antioxidant, and antibacterial effects. This study conducted a comparative analysis of the antitumor activities and underlying molecular mechanisms of two *Phylloporia* species, *Phylloporia crataegi* and *Phylloporia fontanesiae*, from Shandong Province. *In vitro* assays demonstrated that ethanol extract from the two species exhibited significant, time- and concentration-dependent inhibition of breast cancer (MCF-7), esophageal cancer (Eca-109), and human non-small cell lung cancer (A549). Notably, the ethanol extract of *P. crataegi* (PCCT) displayed the most potent antitumor activity, with the highest inhibition of Eca-109 cells, achieving an IC_50_ of 1.310 ± 0.052 mg/ml. Metabolomic analysis revealed that *P. crataegi* contained significantly higher levels of antitumor-associated metabolites, including trans-cinnamic acid, AMP, and 6-phospho-α-D-glucan (*P* < 0.05). Transcriptomic analysis further indicated that genes involved in cellular stress responses and metabolic pathways were significantly upregulated in *P. crataegi*, including IRE1-mediated unfolded protein responses, ATPase activity, and calcium signaling pathways, contributing to its antitumor properties. Proteomic analysis corroborated these findings, identifying differentially expressed proteins predominantly associated with secondary metabolite biosynthesis and energy metabolism. This study elucidates the molecular mechanisms underlying the superior antitumor activity of *P. crataegi*, underscoring the potential of *Phylloporia* species as valuable resources for drug discovery and therapeutic development.

## Introduction

China is the largest global producer and consumer of medicinal fungi [1], with these organisms emerging as a central focus of mycological research in recent years. Wood-decay fungi, in particular, have demonstrated significant therapeutic potential, surpassing many plant-based and animal-based medicines [2]. Among these, fungi belonging to the *Phylloporia* genus have been recognized for their significant medicinal value. *Phylloporia crataegi* and *Phylloporia fontanesiae* are classified within the Basidiomycota, Hymenomycetes, Aphyllophorales, Hymenochaetaceae and *Phylloporia* [3]. Relevant studies have shown that species within the *Phylloporia* genus, especially *Phylloporia lonicerae*, exhibit a wide range of bioactivities, including antitumor, immune-modulatory, anti-inflammatory, antiviral, antioxidant, and antibacterial effects [4-8]. *Phylloporia* species are also rich in nutrients, non-toxic, and have potential applications in treating tumors, diabetes, AIDS, viral infections, and bacterial growth inhibition [9-11]. However, research has predominantly focused on *P. lonicerae*, while the exploration of *P. crataegi* and *P. fontanesiae* remains relatively limited, and further investigation is urgently required to address this gap.

Several medicinal wood-decay fungi, including *Ganoderma lucidum* [12, 13], *Sanghuangporus* species [14-17], and *Auricularia heimuer* [18, 19], have undergone genomic and transcriptomic analyses. Sequencing of *G. lucidum* revealed that the differentiation of secondary metabolite synthesis gene clusters is often associated with the emergence of novel medicinal activities [12]. Omics research on *Sanghuangporus sanghuang* identified a flavonoid synthesis gene cluster composed of seven core genes. Transcriptomic data indicated differential expression of secondary metabolite biosynthesis gene clusters, with higher production of flavonoids and polysaccharides during the mycelial stage compared to the fruiting body stage [14]. These findings highlight the critical role of omics technologies in elucidating the regulatory mechanisms underlying secondary metabolite biosynthesis in wood-decay fungi and their contribution to medicinal properties. By analyzing dynamic changes in gene expression and metabolite accumulation, researchers can gain deeper insights into the biosynthetic pathways of medicinal fungi, thereby facilitating more effective exploration and utilization of their therapeutic potential. However, compared to other medicinal wood-decay fungi, *P. crataegi* and *P. fontanesiae* remain underexplored, with a notable paucity of omics data.

This study investigated the antitumor properties of the mycelium of *P. crataegi* and *P. fontanesiae* at an identical fermentation stage. Comprehensive multi-omics analyses, including transcriptomics, proteomics, and metabolomics, were conducted to compare transcriptional profiles, protein expression, and secondary metabolite production between the two species. By integrating antitumor activity data with the results of these analyses, the study provides insights into how these molecular differences influence the pharmacological properties of the two species and elucidates the underlying mechanisms responsible for these variations.

## Materials and Methods

### Sample Collection

The wild *P. crataegi* and *P. fontanesiae* strain were isolated and preserved by Shandong Province the Key Laboratory of Applied Mycology, with preservation numbers 554 and 547, respectively.

Human breast cancer cells MCF-7 (TCHu 74), Human non-small cell lung cancer cell line A549 (TCHu 150) were purchased from the cell bank of the Chinese Academy of Sciences, and esophageal cancer cell line Eca-109 (iCell-h056) was purchased from Cybertron Biotechnology Co., Ltd., (China).

### Reagents

MTT, 5-fluorouracil, and apoptosis assay kits were purchased from Beijing Solaibao Technology Co., Ltd.,(China); F-12K, MEM, RPMI-1640, and fetal bovine serum were purchased from Shanghai Xiaopeng Biotechnology Co., Ltd.,(China); PBS buffer, penicillin streptomycin nystatin solution, and trypsin were purchased from Biological Industries; Dimethyl sulfoxide solution were purchased from Tianjin Fuyu Fine Chemical Co., Ltd.,(China).

### Preparation of Sample

**Mycelium extract preparation.** Activated *P. crataegi* and *P. fontanesiae* were transferred to liquid medium for cultivation and subsequently expanded [20]. After cultivation, the mycelium was harvested by filtration and lyophilized. For every 10 g of mycelium powder, 100 ml of 70% ethanol was added and sonicated for 1 h. The mixture was centrifuged at 5,000 rpm for 10 min, and the supernatant was then collected. This extraction was repeated twice, and the combined supernatants were concentrated by rotary evaporation to obtain the ethanol extracts (PCCT and PFCT). The remaining mycelium was mixed with 100 ml of distilled water per 10 g, heated in boiling water for 1 h per extraction, and extracted three times. The combined supernatants were concentrated by rotary evaporation to obtain intracellular aqueous extracts (PCST and PFST).All extracts were lyophilized and stored at 4°C [21].

**Fermentation broth extract preparation.** After filtration of the mycelium, the remaining fermentation supernatant was concentrated to one-tenth of its original volume, mixed with 95% ethanol (4:1, v/v), and allowed to settle for 24 h. After centrifugation (5,000 rpm, 10 min), the precipitates were dissolved in water to obtain ethanol-precipitated extracts (PCCC and PFCC), and the supernatants were rotary evaporated to obtain ethanol-soluble extracts (PCCR and PFCR). All extracts were lyophilized and stored at 4°C.

### *In Vitro* Antitumor Activity Assay

**Cell culture and treatment.** MCF-7, A549, and Eca-109 cells were cultured in MEM, F-12K and RPMI-1640 media, with 10% FBS as the complete medium, at 37°C, 5% CO_2_, and 90% relative humidity. The cells were seeded into 96-well plates for culture. Five concentrations of 62.5, 12.5, 2.5, 0.5 and 0.1 mg/ml were selected, and the cells were treated with 100 ml samples for 24, 48, 72 and 96 h, respectively. The sample solution was then removed.

**Cell proliferation inhibition.** The MTT assay was used to determine the cell proliferation inhibition rate [22], with 5-fluorouracil as the positive control [23]; each well of the plate was added 20 μl of 5 mg/ml MTT and 80 μl of the corresponding complete culture medium, and incubated at 37°C in a 5% CO_2_ incubator for 4 h. The mixture was removed, and 150 μl of dimethyl sulfoxide was added to dissolve the purple formazan crystals. The plate was shaken and mixed for 20 min, and the absorbance was measured at 490 nm. The inhibition rate was calculated using the following formula:

$$\text{Inhibition Rate }\left(\%\right)=\left(1-\frac{\text{OD sample group}}{\text{OD negative control}}\right)\times 100$$

### Multi-Omics Analysis

The analysis was performed at PANOMIK Biopharmaceutical Technology Co., Ltd., Suzhou. Cultures fermented for 5 days were centrifuged at 5,000 rpm and 4°C for 5 min, and the supernatant was discarded. Cultures fermented for 5 days were centrifuged at 5,000 rpm and 4°C for 5 min, and the supernatant was discarded. The precipitates were snap-frozen in liquid nitrogen for 1 min and stored at -80°C.

**Untargeted metabolomics analysis.**

**Sample preparation and LC-MS analysis.** LC analysis was performed on a Vanquish UHPLC system and an ACQUITY UPLC HSS T3 (150 × 2.1 mm, 1.8 μm) column. The flow rate and injection volume were set at 0.25 ml/min and 2 μl, respectively. For LC-ESI (+)-MS analysis, the mobile phases consisted of (B2) 0.1% formic acid in acetonitrile (v/v) and (A2) 0.1% formic acid in water (v/v). Separation was conducted under the following gradient: 0-1 min, 2% B2; 1-9 min, 2%-50% B2; 9-12 min, 50%-98% B2; 12-13.5 min, 98% B2; 13.5-14 min, 98%-2% B2; 14-20 min, 2% B2. For LC-ESI (-)-MS analysis, the analytes was carried out with (B3) acetonitrile and (A3) ammonium formate (5 mM). Separation was conducted under the following gradient: 0-1 min, 2% B3; 1-9 min, 2%-50% B3; 9-12 min, 50%-98% B3; 12-13.5 min, 98% B3; 13.5-14 min, 98%-2% B3; 14-17 min, 2% B3 [24].

**Mass spectrum conditions and data processing.** Mass spectrometric detection of metabolites was performed on Orbitrap Exploris 120 (Thermo Fisher Scientific, USA) with ESI ion source. Simultaneous MS1 and MS/MS (Full MS-ddMS2 mode, data-dependent MS/MS) acquisition was used. The parameters were as follows: sheath gas pressure, 30 arb; aux gas flow, 10 arb; spray voltage, 3.50 kV and -2.50 kV for ESI (+) and ESI (-), respectively; capillary temperature, 325°C; MS1 range, m/z 100-1000; MS1 resolving power, 60000 FWHM; number of data dependant scans per cycle, 4; MS/MS resolving power, 15000 FWHM; normalized collision energy, 30%; dynamic exclusion time, automatic [25].

Metabolites quantitative analysis was conducted using the R XCMS (version 3.12.0) package. The identification of metabolites was performed using public databases (HMDB, MassBank, LipidMaps, mzCloud, KEGG) and an in-house library. PCA, PLS-DA, and OPLS-DA models were validated through permutation tests. Functional pathway enrichment and topological analysis of differential metabolites were conducted using MetaboAnalyst (version 5.0) software, with KEGG Mapper (version 5.2) tools for pathway visualization.

**Non-referenced transcriptomics analysis.** RNA purity and concentration were measured using a Nanodrop spectrophotometer. Library were assessed with an Agilent 2100 Bioanalyzer [26]. RNA was extracted from mycelium using a commercial kit. Its quality and concentration were assessed using Nanodrop and agarose gel electrophoresis, followed by sequenced on Illumina HiSeq (China). Sequences obtained from the transcriptome were used to design qRT-PCR primers. Relative quantification was performed using SYBR GREEN dye, with 18S rRNA as the internal control. qRT-PCR conditions were as follows: 95°C for 5 min (1 cycle); 95°C for 10 sec, 60°C for 30 sec (40 cycles). Melting Curve: 95°C for 15 sec, 60°C for 60 sec, 95°C for 15 sec (continuous detection). Raw sequencing files were deposited to NCBI (SRA, https://www.ncbi.nlm.nih.gov/sra, Bioproject Accession: PRJNA1271313, SRA Accession: SRR33802027 to SRR33802038).

**Proteomic analysis.**

**Protein extraction and digestion.** Proteins were extracted via acetone precipitation and quantified using the BCA assay [27]. Proteins were digested with trypsin, following reduction with DTT and alkylation with IAA. The reaction was terminated by the addition of formic acid, and the peptides were desalted using C18 columns, subsequently dried for HPLC analysis [28].

**LC-MS/MS Analysis.** Dried peptides were analyzed using an Orbitrap Eclipse mass spectrometer, equipped with a Nanospray Flex ion source and FAIMS Pro Interface. Chromatographic separation was achieved using a gradient of 0.1% formic acid in water (solvent A) and 80% acetonitrile containing 0.1% formic acid (solvent B). Mass spectrometric analysis was performed using an ORBITRAP ECLIPSE mass spectrometer, which was equipped with a FAIMS Pro Interface. Data acquisition followed a data-dependent acquisition (DDA) strategy [29, 30].

**Data processing.** Proteomic data were processed using Proteome Discoverer 2.4, with alignment against the Hymenochaetaceae database. RNA sequencing was performed using an Illumina sequencing platform. Gene expression levels were quantified using RSEM, and differentially expressed genes (DEGs) were identified with DESeq2, with thresholds of |log2FC| ≥ 1 and FDR < 0.05. GO and KEGG enrichment analyses of DEGs were conducted using tools such as clusterProfiler (version 4.4.4). The mass spectrometry proteomics data generated in this study have been deposited to the Proteome Xchange Consortium via the iProX partner repository with the dataset identifier PXD064771 (https://proteomecentral.proteomexchange.org) [31, 32].

### Molecular Docking Analysis

The canonical SMILES of the top fifteen differential metabolites were retrieved in PubChem (https://pubchem.ncbi.nlm.nih.gov/). Corresponding target proteins were identified using the Swiss Target Prediction database (https://www.swisstargetprediction.ch/), with a prediction probability greater than 0. Esophageal cancer, non-small cell lung cancer, breast cancer, and malignant neoplasm of esophagus were used as keywords to query target proteins through OMIM (https://www.omim.org/) and GeneCards (https://www.genecards.org/). The names of the target proteins were standardized using the NCBI and UniProt databases (https://www.uniprot.org/). The intersection of three cancer-related targets was selected for further analysis.

To investigate the role of SYM in cancer treatment, a protein-protein interaction (PPI) network of intersecting human genes was constructed using the STRING database (https://cn.string-db.org/), with Homo sapiens as the species and a Minimum Required Interaction Score of 0.4. The network was visualized by Cytoscape 3.7.2, and Degree Centrality (DC), Betweenness Centrality (BC), and Closeness Centrality (CC) of the nodes were analyzed using Centiscape 2.2. Core targets were identified based on median values of all three centrality parameters.

Molecular structure files of the compounds were obtained from PubChem and energy-minimized using Chem3D (version 23.1.1.3). The 3D crystal structures of the key target proteins were downloaded from the RCSB PDB database (https://www.rcsb.org/). The target proteins and compounds were prepared using PyMOL (version 2.6.0a0) and Autodock Tools (version 1.5.7). Blind docking was performed to identify the active site[33], and virtual docking was then carried out using AutoDock Vina (version 1.1.2) to evaluate ligand-receptor interactions.

### Statistical Analysis

The IC_50_ values of the sample solutions were calculated and plotted using GraphPad Prism 9 statistical software. A *P*-value of <0.01 was considered to indicate highly significant statistical differences.

## Results

### *In Vitro* Antitumor Activity of Extracts from Two Species of *Phylloporia*

Table 1 presents the *in vitro* IC_50_ values of extracts from *Phylloporia* species against three tumor cell lines (A549, MCF-7, and Eca-109). The results indicate that the extracts from both *Phylloporia* species exhibit varying degrees of inhibitory effects on the proliferation of the three tumor cell lines. Among these, the ethanol extract of *P. crataegi* (PCCT) showed the most potent antitumor activity, exhibiting the highest inhibition of Eca-109 cells with an IC_50_ of 1.310 ± 0.052 mg/ml. Additionally, significant inhibitory effects were observed in A549 and MCF-7 cells, with IC_50_ values of 2.300 ± 0.128 mg/ml and 3.520 ± 0.110 mg/ml, respectively, which were superior to *P. fontanesiae*.

### Untargeted Metabolomics Analysis

**Fig. 1:**
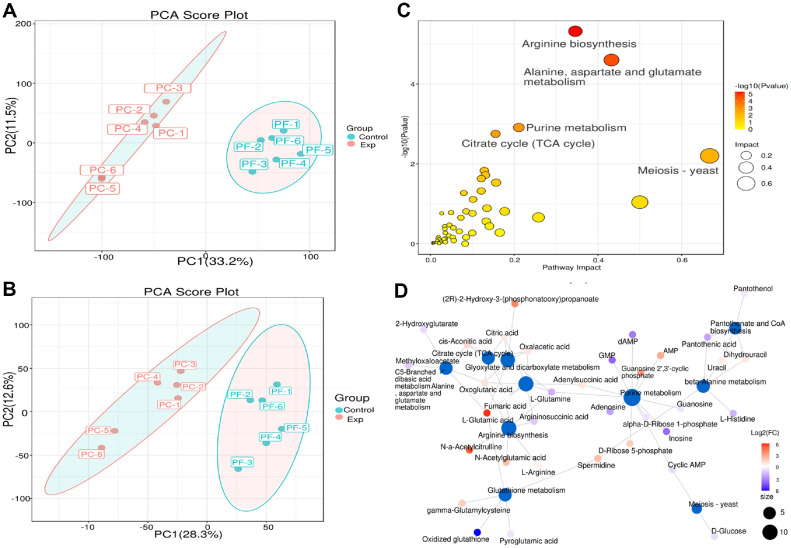
(A) Principal component analysis plots (ESI+). (B) Annotated network diagram of differential metabolites (ESI-). (C) Bubble diagram of metabolic pathway impact factors. (D) Annotated network diagram of differential metabolites.

**Principal component analysis (PCA).** As shown in Fig. 1 (A-B), in the positive ion mode (ESI+), PC1 and PC2 accounted for 33.2% and 11.5% of the variance, respectively. In the negative ion mode (ESI-), PC1 and PC2 explained 28.3% and 12.6%, respectively. Samples within the same group clustered together, while samples between groups were widely dispersed. This pattern suggests significant differences between the groups and no significant difference within the group, further supporting the repeatability and reliability of the results.

**Identification of Differential Metabolites (DAMs) - MS/MS.** Metabolites were annotated based on MS/MS fragment patterns and accurate molecular weights using several databases, including the Human Metabolome Database (HMDB), MassBank, LipidMaps, mzCloud, and the Nominee Metabolism Standard Database. Differential metabolites were selected from the primary metabolite list according to screening criteria of *P*-value <0.05 and VIP threshold>1. As shown in Table 2, seventeen metabolites exhibited a differential fold change greater than 5. The statistical significance, represented by the -log_10_ transformed P-values, ranged from 1 to 12.11.

Notably, the concentration of diaminoheptanedioic acid in *P. crataegi* was 310.13-fold higher compared to that in *P. fontanesiae*. Other metabolites that exhibited significant differences include L-glutamic acid, trans-cinnamic acid, and N-α-acetylated lysine.

**Differential metabolite pathway analysis.** KEGG annotation and enrichment analyses were conducted to investigate the functions and associated biological processes of differential abundant metabolites (DAMs).

As shown in Fig. 1 (C-D), differential metabolites significantly affect the arginine biosynthesis pathway, as well as the alanine, aspartate, and glutamate metabolic pathways. These amino acid metabolism and synthesis pathways are closely related to protein synthesis, with a notable enrichment of differentially expressed metabolites.

### Unreferenced Transcriptomics Analysis

**Fig. 2:**
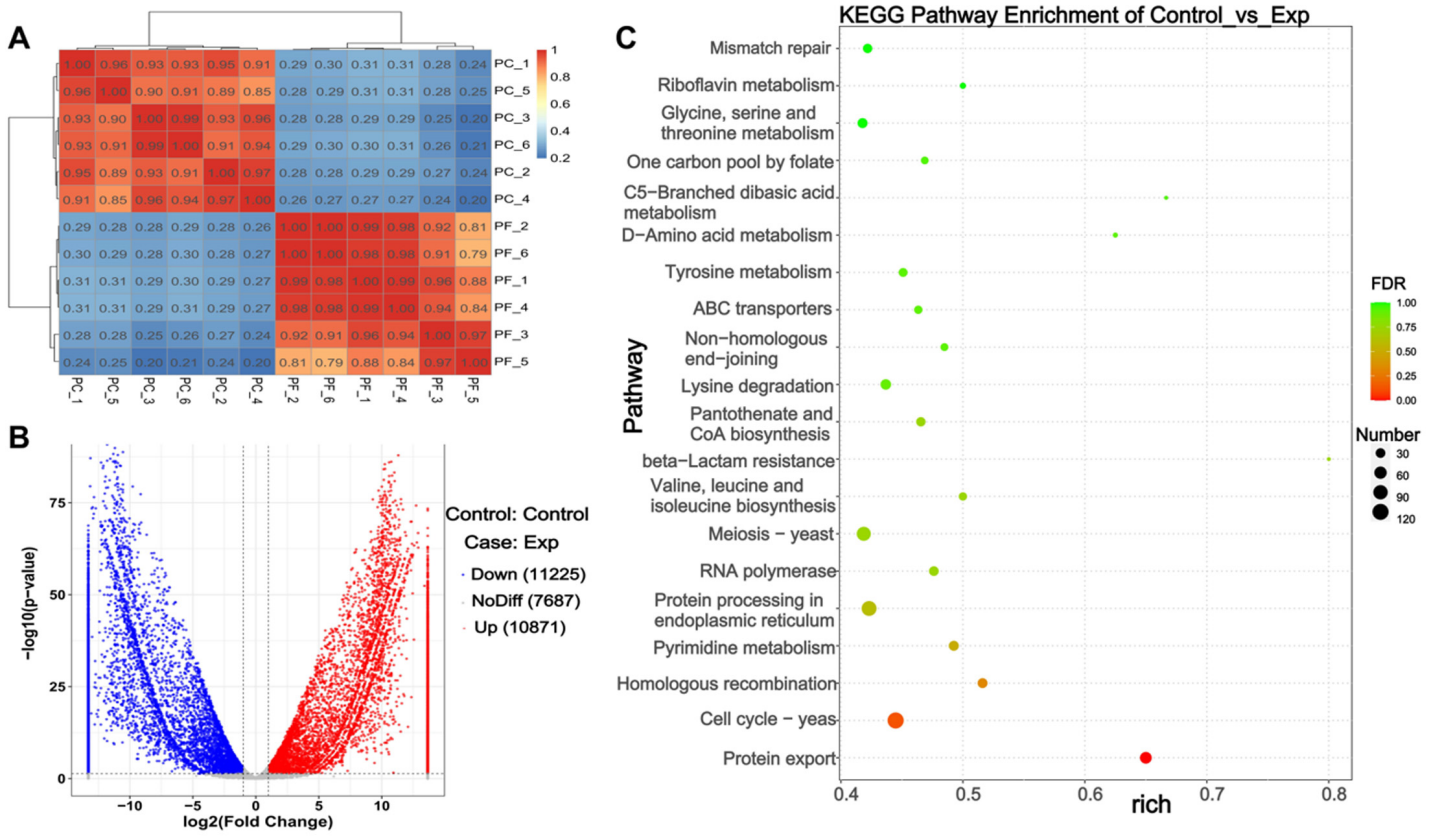
PC represents *P. crataegi* samples (Exp group), and PF represents *P. fontanesiae* samples (Control group), with three biological replicates for each. (**A**) Relative gene expression of differential transcripts. (**B**) Gene expression correlation. (**C**) Volcano plot of differentially expressed genes.

**Transcriptome correlation analysis.** The correlation coefficient of gene expression levels within the same sample group approached 1, with a correlation coefficient of 1 for identical samples, indicating excellent reproducibility and a strong correlation within the group. The correlation coefficient of gene expression levels between different groups was below 0.8, suggesting significant differences between groups. This ensures the reliability of the experiment, justifies sample selection, and fulfills the criteria for differential transcript analysis. As shown in Fig. 2A, differentially expressed genes were identified using DESeq analysis, with thresholds of *P*-value < 0.05 and a log2 fold change > 1. As illustrated in Fig. 2B, a total of 22,096 differentially expressed genes were identified, with 11,225 upregulated and 10,871 downregulated.

**GO Annotation and enrichment analysis.** As shown in Table 3, differentially expressed genes enriched in cellular components such as the endoplasmic reticulum and nucleus, influence molecular functions such as ATPase activity, voltage-gated calcium channel activity, aspartate carbamoyltransferase activity, tubulin binding, and affect biological processes like the IRE1-mediated unfolded protein response pathway.

**KEGG annotation and enrichment analysis.** As shown in Fig. 2C, within the category of Cellular Processes, differential expressed genes were significantly enriched in cell cycle and meiosis. The expression of genes related to ABC transporters was significantly upregulated in the *P. crataegi* group compared to the control group. Pyrimidine metabolism was the most significant enriched pathway in the metabolism category, and was followed by pantothenate and CoA biosynthesis and multiple amino acid anabolic pathways. In the category of genetic information processing, differential genes were significantly enriched in protein export and homologous recombination, protein processing in endoplasmic reticulum and RNA polymerase.

### Label-Free Proteomics Analysis

**Fig. 3:**
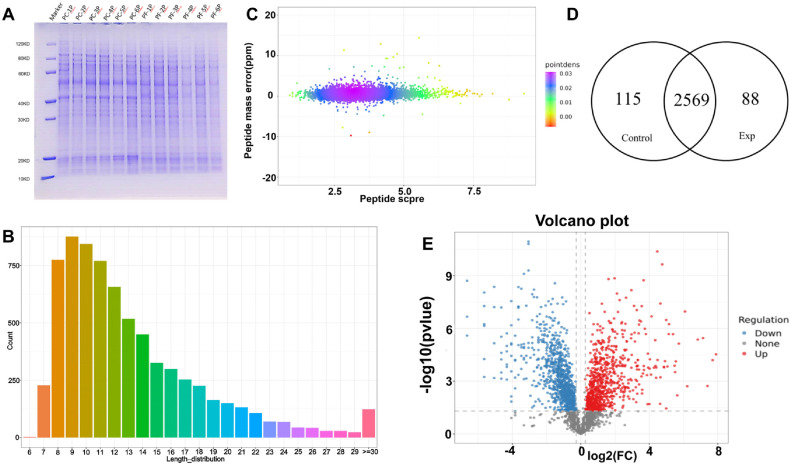
(A) SDS-PAGE gel electrophoresis of protein samples. (B) Peptide segment length distribution. (C) Peptide mass shift. The color in the graph indicates the density distribution of scatter points. (D) Venn diagram of identified protein recombination. (E) Volcano plot of differentially expressed proteins.

**Validation of differential transcripts by quantitative PCR.** As shown in Fig. 3A, the protein bands are clear, complete, uniform, stable, and non-degraded, meeting the requirements of this study. Quality control of mass spectrometry results are displayed in Fig. 3B-3C, indicating that the mass deviation was mainly within ±20 ppm, and peptide lengths ranged from 8 to 35 amino acid residues. The overall protein sequence coverage was high, indicating that sample preparation met quality standards and trypsin digestion was complete, thus providing robust data for downstream quantitative and functional studies.

**Protein identification analysis.** Quality control processing was performed on the data using the criteria of Unique Peptide ≥ 1 and proteins quantified in at least 50% of the samples. As shown in Fig. 3D, 2,684 proteins were identified in the Control group (*P. fontanesiae* samples) and 2,657 proteins in the Exp group (*P. crataegi* samples). Among them, 2,569 proteins were commonly identified in both groups, with 115 specific to *P. fontanesiae* and 88 specific to *P. crataegi*, indicating differences in protein expression between the two *Phylloporia* species.

**Differential protein screening.** As shown in Fig. 3E, based on the significance of differential expression between two samples (*P*-value < 0.05 and fold change (FC) > 1.2 for upregulation (log2 (FC) > 0) and FC < 1.2 for downregulation (log2 (FC) < 0), a total of 1,694 differentially expressed proteins were identified, of which 924 were downregulated and 770 upregulated.

**GO and KEGG enrichment analysis.** A total of 157 enriched GO terms were identified, with the top 20 most significantly enriched functional classifications (*P*-value < 0.05) displayed in bar charts. Differentially expressed proteins were analyzed across biological processes, cellular components, and molecular functions, and were subsequently ranked by significance. As shown in Table 4, the differential expressed proteins were predominantly involved in biological processes such as protein transport, carbohydrate metabolism, and the tricarboxylic acid cycle. They were primarily localized to the membrane, cytoplasm, and nucleus, and their major molecular functions included metal ion binding, oxidoreductase activity, and ATP binding.

**Fig. 4:**
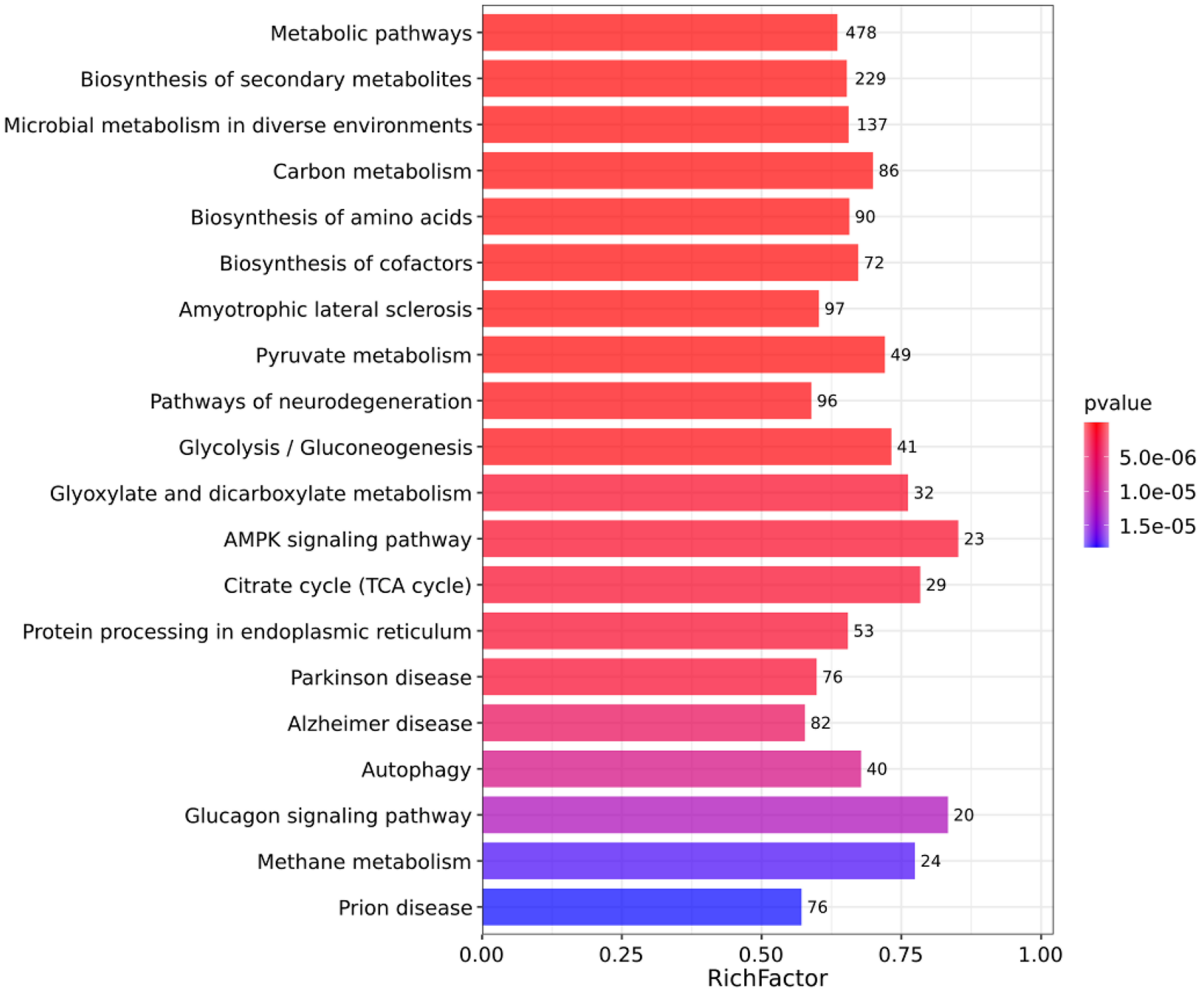
Top 20 bar plot of KEGG pathway enrichment.

As shown in Fig. 4, KEGG pathway enrichment analysis identified 144 pathways, of which the top 20 most significantly enriched pathways (*P* value < 0.05) including metabolic pathways, amino acid biosynthesis, secondary metabolite biosynthesis, carbon metabolism, among others, which isconsistent with the enrichment results obtained from transcriptomics and metabolomics analyses.

**Fig. 5:**
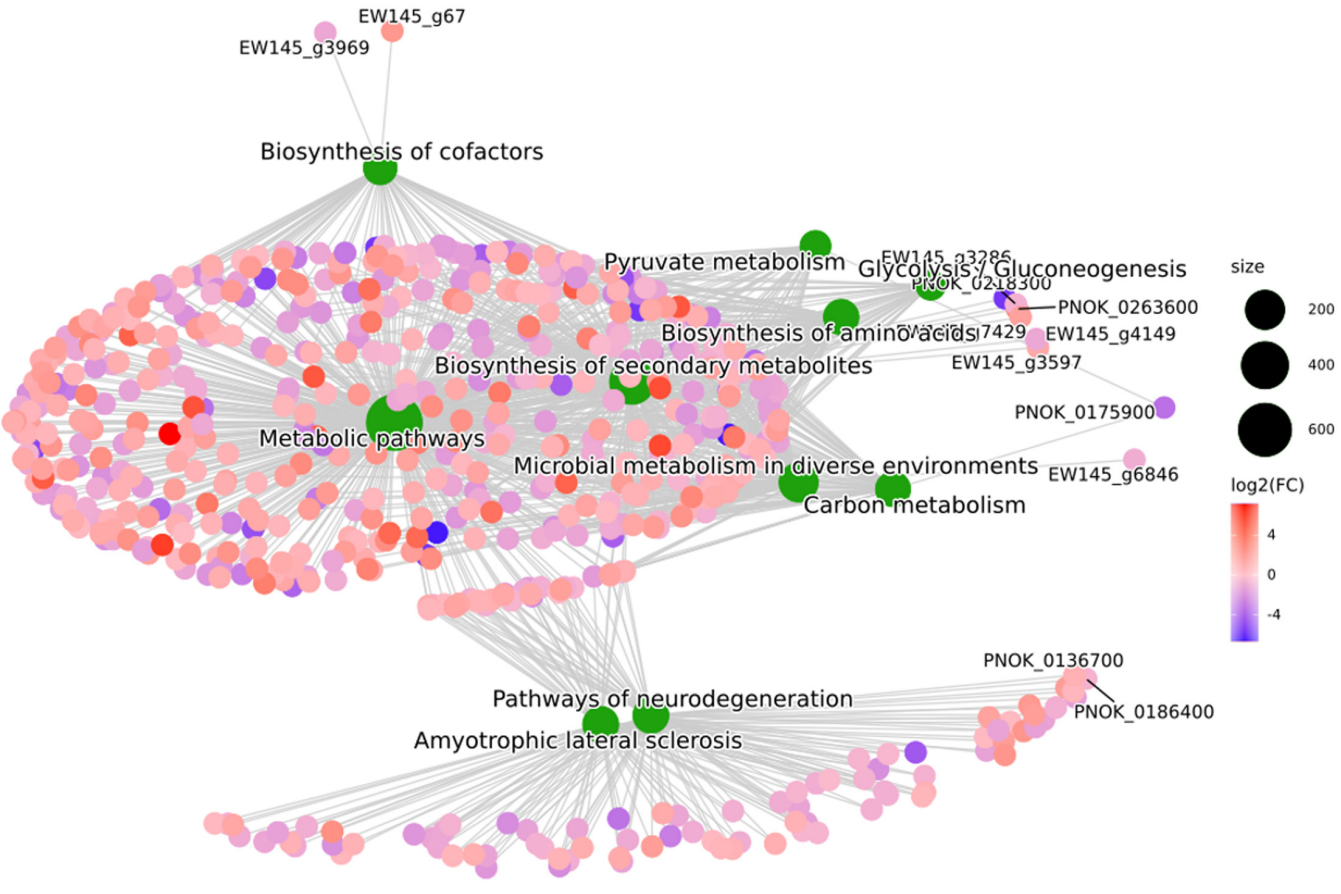
Pathway enrichment map of differentially expressed proteins.

**Protein interaction network analysis.** As shown in Fig. 5, differentially expressed proteins were significantly enriched in pathways such as secondary metabolite biosynthesis, metabolic pathways, microbial metabolism in diverse environments, amino acid biosynthesis, carbon metabolism, cofactor biosynthesis, pyruvate metabolism, and glycolysis. Differences in pharmacological activities of the two leaf pore bacteria may be due to the interaction and coordination of the differential proteins triggering the production of differential metabolites.

### Molecular Docking of Key Metabolites

**Fig. 6:**
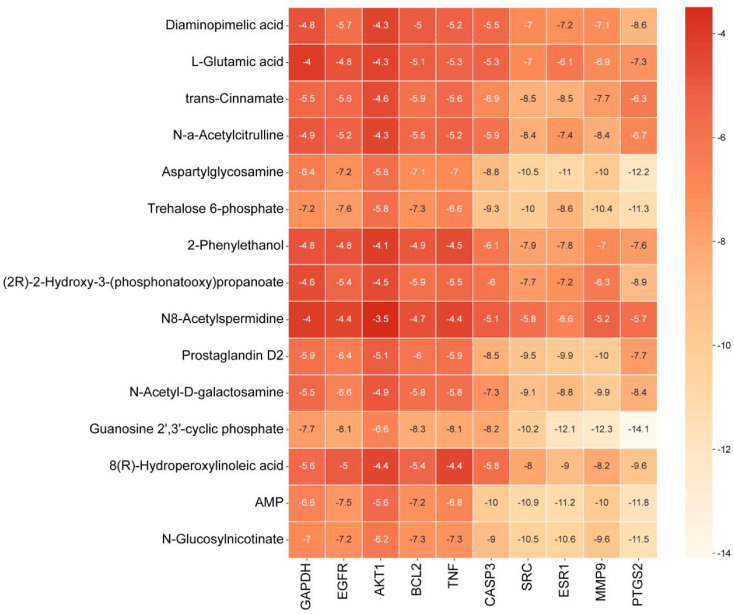
Molecular docking binding energy results.

To investigate the molecular mechanisms of the antitumor activity of *P. crataegi* and *P. fontanesiae*, molecular docking studies were conducted to assess the binding affinities of the differential metabolites to cancer-related therapeutic targets. The selection of core targets was based on the criterion that all three parameters exceed the median. Initially, 81 nodes and 1,120 edges were identified, which were reduced to 22 nodes and 209 edges following the screening process. The top ten core targets for the study were selected based on degree centrality, as shown in Table 5. A binding energy < 0 kcal/mol indicates that the compound can freely bind to the docked target protein, and a binding energy < -5 kcal/mol indicates that the compound binds well to the target protein [34]. As demonstrated in Fig. 6, all differential metabolites show binding affinity for the core target proteins, with multiple differential metabolites exhibiting strong binding interactions. These key target proteins are involved in in a variety of cancer-associated pathways, and thus, the differential metabolites may exert anticancer therapeutic effects by modulating these targets. In particular, Guanosine 2',3'-cyclic phosphate demonstrated exceptional binding activity to multiple docked target proteins, exhibiting the strongest binding affinity to PTGS2 (-14.1 kcal/mol), suggesting its potential significance in cancer therapy.

## Discussion

Research has demonstrated that numerous secondary metabolites derived from medicinal wood-decaying fungi possess diverse bioactivities, including anticancer, anti-inflammatory, antibacterial, antiviral, and other physiological effects [35]. As Outlined in the introduction, the genus *Phylloporia*, a group of medicinal wood-decaying fungi, has demonstrated various pharmacological activities, including anticancer, immune-enhancing, and anti-inflammatory effects. Given these pharmacological activities, increasing attention has been devoted to exploring their chemical constituents. Notably, most studies on the genus *Phylloporia* have focused on *Phylloporia* ribis [8, 21, 36, 37].

This study provides the first comparative multi-omics analysis of the antitumor mechanisms underlying two relatively understudied wood-decay fungi, *P. crataegi* and *P. fontanesiae*. Our findings demonstrate that the ethanol extract of *P. crataegi* exhibits significantly stronger inhibitory effects on tumor cell proliferation compared to *P. fontanesiae*, particularly against esophageal cancer (Eca-109) cells. To elucidate the molecular mechanisms underlying these differences, we conducted comprehensive metabolomic, transcriptomic, and proteomic analyses.

Metabolomic analysis revealed that the differences in pharmacological activities between two wood-decaying fungi, *P. crataegi* and *P. fontanesiae*, are primarily attributed to variations in the content of carboxylic acid metabolites and their derivatives. *P. crataegi* samples exhibited significantly higher levels of key metabolites, such as trans-cinnamic acid, AMP, and trehalose 6-phosphate, compared to those of *P. fontanesiae*. These metabolites are crucial in terms of antioxidant and immunomodulatory effects, as well as in inhibiting tumor cell proliferation. The remarkably high abundance of trans-cinnamic acid in *P. crataegi* (60.86-fold higher than *P. fontanesiae*) likely plays a central role in its superior pharmacological properties. Trans-cinnamic acid is a well-established inhibitor of tyrosinase, an enzyme essential for melanin synthesis [38]. It also inhibits the proliferation of and induces apoptosis in non-small cell lung cancer (NSCLC) and liver cancer cells, acting as an effective inhibitor of both NSCLC and human pancreatic cancer cells (A549-1) [39]. Moreover, trans-cinnamic acid and spermidine stimulate the rapid production and activation of lymphocytes, enhancing immune function by reducing immune-suppressive cell activities, thus exhibiting significant immune-enhancing effects [40-44]. Furthermore, *P. crataegi* exhibited an 18.09-fold higher concentration of trehalose 6-phosphate and a 7.07-fold higher concentration of AMP compared to *P. fontanesiae*. Trehalose 6-phosphate has demonstrated notable antibacterial and anti-inflammatory properties, contributing to metabolic enhancement and protection against viral diseases, particularly in liver health. AMP-activated protein kinase (AMPK) plays a critical role in cellular energy metabolism and homeostasis. Numerous studies have indicated that the AMPK signaling pathway inhibits the proliferation of various tumor cells [45-47]. Protein synthesis is a key process for cell growth and development. Pathway enrichment analysis highlights that both *P. crataegi* and *P. fontanesiae* affect key metabolic processes such as arginine biosynthesis, alanine, aspartate and glutamate metabolism, thereby influencing various biological functions and pharmacological activities for tumor cell proliferation and survival.

Transcriptome analysis reveals up-regulation of genes in *P. crataegi* associated with the endoplasmic reticulum (ER) and the nucleus, an important site for protein folding and quality control. The nucleus is an important site for the storage, replication and transcription of genetic information, as well as a regulatory center for cell growth, division and metabolic activities. The enhancement of its function may help *P. crataegi* to better fulfill its anticancer role. Notably, the IRE1-mediated unfolded protein response (UPR) pathway, a critical regulator of cellular stress, has been implicated in various cancers. The unfolded protein response (UPR) is a complex signaling network that primarily serves as an adaptive mechanism, helping cells restore endoplasmic reticulum (ER) homeostasis and survive [48]. However, excessive or prolonged activation of the UPR can lead to the initiation of apoptotic pathways [49]. Interestingly, IRE1α overexpression or activation in tumor cells has been shown to inhibit tumor growth in immunocompetent mice, highlighting its potential role in enhancing antitumor immune responses [50]. In *P. crataegi*, heightened activity of the UPR-particularly through the IRE1α pathway-may accelerate the progression of ER stress in cancer cells beyond a critical threshold, thereby initiating apoptotic signaling [51]. At the molecular level, upregulated ATPase activity and voltage-gated calcium channel activity in *P. crataegi* could disrupt energy metabolism and calcium homeostasis in tumor cells. ATPases play a crucial role in maintaining ion gradients and cellular energetics, while calcium signaling is integral to regulating apoptosis and cell proliferation [52]. Simultaneously, enhanced tubulin binding activity could interfere with microtubule dynamics, impairing mitosis and metastasis in cancer cells [53]. In summary, these findings highlight the anticancer potential of *P. crataegi* through multiple molecular mechanisms.

Proteomics analysis further revealed significant changes in protein expression under different conditions, particularly within metabolic pathways, secondary metabolite biosynthesis and microbial metabolism. The proteomics data were consistent with the findings from transcriptomics and metabolomics analyses, identifying multiple differentially expressed proteins involved in protein translocation, sugar metabolism, and the tricarboxylic acid cycle (TCA). These differentially expressed proteins were enriched in several metabolic pathways such as secondary metabolite biosynthesis, carbon metabolism, and cofactor biosynthesis. This enrichment further confirming the metabolic pathway enrichment observed in transcriptomics and metabolomics, and highlighting the critical role of metabolism in regulating biological functions. In addition, protein interaction network analyses suggest that the coordinated action of differential proteins may be critical for initiating the synthesis of biologically active secondary metabolites. These secondary metabolites may significantly contribute to the antitumor activity of the fungus, particularly by influencing tumor cell proliferation, apoptosis, and immune response. Therefore, these findings provide some basis for further exploring the potential and mechanism of action of *P. crataegi* and *P. fontanesiae* in tumor therapy.

Molecular docking results demonstrate strong binding affinities between the differential metabolites and key targets such as GAPDH, CASP3, and SRC, which are essential in regulating cellular processes like proliferation and apoptosis. Trans-cinnamate demonstrated strong binding affinities for SRC and ESR1 (-8.5 kcal/mol), suggesting that trans-cinnamate may promote cancer cell death through modulation of key signaling pathways involved in cell survival and apoptosis. Additionally, AMP shows strong binding affinities with AKT1 (-5.6 kcal/mol) and SRC (-10.9 kcal/mol), indicating its potential to regulate cellular metabolism and promote apoptosis through the AMPK pathway. Guanosine 2',3'-cyclic phosphate exhibits strong binding affinity with PTGS2 (-14.1 kcal/mol), suggesting that it may exert antitumor effects by inhibiting inflammatory pathways and activating guanosine-mediated tissue protection mechanisms.

By integrating metabolomic, transcriptomic, proteomic, and molecular docking analyses, this study provides a comprehensive understanding of the molecular mechanisms underlying the superior antitumor activity of *P. crataegi* compared to *P. fontanesiae*. Metabolomics data reveal that the accumulation of various differential metabolites in *P. crataegi*, such as trans-cinnamate, AMP, and Guanosine 2',3'-cyclic phosphate, regulates key metabolic and apoptosis pathways to exert anti-tumor effects. Transcriptomics analysis further reveals that differentially expressed genes in *P. crataegi* significantly influence multiple metabolic pathways and the accumulation of related metabolites, such as pyrimidine metabolism, coenzyme A biosynthesis, and amino acid synthesis pathways. Notably, the activation of the IRE1-mediated unfolded protein response (UPR) pathway may regulate the activity of key metabolic pathways, promote the accumulation of metabolites, enhance the endoplasmic reticulum stress response, and drive apoptosis of cancer cells through pro-apoptotic mechanisms. The interaction between the transcriptomic and metabolic layers synergistically enhances the antitumor effect of *P. crataegi*. Moreover, proteomics results also show that differentially expressed proteins are significantly enriched in multiple key metabolic pathways, such as metabolic pathways, amino acid biosynthesis, secondary metabolite biosynthesis, and carbon metabolism, which align closely with the enrichments observed in the transcriptomics and metabolomics data. For instance, in amino acid biosynthesis, the regulation of differential proteins coordinates with the changes in gene expression observed in the transcriptome and is closely associated with the accumulation of metabolites like L-Glutamic acid, AMP, and N-a-Acetylcitrulline. To further validate the role of these metabolites in the antitumor effect, we conducted molecular docking studies to explore the interactions between differential metabolites in *P. crataegi* and core tumor targets such as SRC, GAPDH, and CASP3. The molecular docking results indicate that these metabolites can bind freely with the core targets, and most of them show strong binding affinities, providing further evidence of their crucial roles in regulating tumor cell proliferation and apoptosis.

In summary, the antitumor activity of *P. crataegi* is not only dependent on the accumulation of differential metabolites but is also intricately regulated through interactions between transcriptomics and proteomics. The molecular docking studies further confirm the strong binding affinity between key metabolites and tumor targets, highlighting their crucial roles in regulating tumor cell proliferation and apoptosis. This offer solid molecular-level evidence supporting the antitumor mechanism of *P. crataegi* and providing a theoretical foundation for future drug development.

These findings provide a comprehensive understanding of the molecular basis underlying the superior antitumor activity of *P. crataegi*, providing valuable insights into its biological functions and pharmacological properties, and contributing to the broader understanding of the *Phylloporia* genus. Furthermore, the secondary metabolites of *Phylloporia* species provide theoretical support for the broader application of medicinal fungi in drug development. The application of fermentation products as substitutes for fruiting bodies and mycelium in medicinal activity research addresses the challenges related to resource scarcity while reducing development costs, thereby enhancing practical feasibility. This study highlights the potential of *Phylloporia* species as a valuable resource for drug discovery and provides a theoretical foundation for exploring its application in drug development.

## Figures and Tables

**Table 1:** *In vitro* antitumor IC_50_ values of extracts from two *Phylloporia* species.

Sample	IC_50_ (mg/ml)		
	A549 (24 h)	MCF-7 (72 h)	Eca-109 (72 h)
PCCC	4.520±0.620^C^	15.710±2.650^B^	12.530±0.188^E^
PCCR	42.180±1.202^BC^	45.830±0.420^A^	22.490±1.351^D^
PCST	5.690±0.648^C^	5.470±1.090^DE^	18.070±0.551^DE^
PCCT	2.300±0.128^C^	3.520±0.110^E^	1.310±0.052^F^
PFCC	87.460±8.135^B^	8.620±0.610^CD^	40.640±5.271^C^
PFCR	169.200±65.914^A^	5.480±3.160^DE^	117.630±6.850^A^
PFST	63.560±3.828^BC^	12.220±0.820^BC^	73.630±2.106^B^
PFCT	5.570±0.091^C^	7.950±1.000^D^	5.700±0.400^F^

These data represent the average IC_50_ values ± standard deviations for different samples across three cell lines (A549, MCF-7, and Eca-109). Different uppercase letters within the same column indicate statistically significant differences in IC_50_ values between samples for a given cell line at the 0.01 level.

**Table 2:** Seventeen metabolites exhibited a differential fold change greater than 5.

Name	FC	Log2FC	-log_10_ (P.value)	VIP
Diaminopimelic acid	310.130	8.280	6.690	1.929
L-Glutamic acid	67.790	6.080	12.110	1.781
trans-Cinnamate	60.860	5.930	2.520	1.382
N-a-Acetylcitrulline	38.360	5.260	1.330	1.105
Aspartylglycosamine	20.960	4.390	3.020	1.645
Trehalose 6-phosphate	18.090	4.180	3.800	1.713
2-Phenylethanol	17.990	4.170	3.070	1.445
(2R)-2-Hydroxy-3-(phosphonatooxy)propanoate	16.000	4.000	2.670	1.677
N8-Acetylspermidine	13.260	3.730	5.250	1.698
Prostaglandin D2	12.260	3.620	3.700	1.572
N-Acetyl-D-galactosamine	10.490	3.390	7.140	1.752
Guanosine 2',3'-cyclic phosphate	9.450	3.240	6.670	1.742
8(R)-Hydroperoxylinoleic acid	7.430	2.890	2.240	1.499
AMP	7.070	2.820	2.620	1.384
N-Glucosylnicotinate	6.580	2.720	2.470	1.485
Tyrosol	5.90	2.560	6.690	1.929
Porphobilinogen	5.30	2.410	12.110	1.781

Name: Identification result; FC: Fold Change, represents the linear expression multiple change of the experimental group relative to the control group; Log2FC: The logarithmic multiple change is the logarithm of FC with base 2, which is used to symmetrize the data for easy comparison and statistical score; P.value: The smaller the difference, the more significant; VIP: OPLS-DA first principal component variable importance projection.

**Table 3:** GO Enrichment analysis of upregulated DEGs between *P. fontanesiae* and *P. Crataegi*.

Category	Term	-log10 (P.value)
CC	Perinuclear endoplasmic reticulum lumen	11.009
CC	Cortical endoplasmic reticulum lumen	11.009
CC	Luminal surveillance complex	10.886
CC	Perinuclear endoplasmic reticulum	7.886
CC	Endoplasmic reticulum lumen	6.367
CC	Nucleus	5.432
BP	IRE1-mediated unfolded protein response	9.721
BP	Karyogamy involved in conjugation with cellular fusion	9.244
BP	Posttranslational protein targeting to membrane, translocation	9.125
BP	Karyogamy	8.097
BP	Posttranslational protein targeting to endoplasmic reticulum membrane	7.620
BP	Nuclear chromosome segregation	9.721
MF	ATPase activity	3.886
MF	Voltage-gated calcium channel activity	3.187
MF	Voltage-gated cation channel activity	2.866
MF	Aspartate carbamoyltransferase activity	2.785
MF	Tubulin binding	2.710
MF	Drug binding	2.625

GO terms in each category (CC, BP, and MF) are ranked by ascending P-value, only the top 6 terms are shown. CC stands for cellular components; BP stands for biological processes; MF stands for molecular functions.

**Table 4:** GO Enrichment Analysis between *P. fontanesiae* and *P. Crataegi*.

Category	Term	Count	-log_10_ (P.value)	RichFactor
CC	Membrane	174	36.535	0.617
CC	Cytoplasm	131	28.796	0.636
CC	Nucleus	126	27.678	0.636
CC	Mitochondrial inner membrane	33	9.829	0.733
CC	Golgi membrane	15	7.379	0.938
BP	Protein transport	38	8.668	0.745
BP	Carbohydrate metabolic process	42	7.491	0.677
BP	Tricarboxylic acid cycle	19	4.767	0.760
BP	Phosphorylation	28	4.661	0.651
BP	Endoplasmic reticulum to Golgi vesicle-mediated transport	13	3.900	0.813
MF	Metal ion binding	135	7.848	0.637
MF	Oxidoreductase activity	67	7.351	0.728
MF	ATP binding	234	5.973	0.560
MF	Small GTPase binding	13	3.586	0.929
MF	Calcium ion binding	25	3.480	0.758

GO terms in each category (CC, BP, and MF) are ranked by ascending P-value, only the top 5 terms are shown. Higher Rich Factor values correspond to more pronounced enrichment.

**Table 5:** Three parameter values for the core target.

Name	Betweenness centrality	Closeness centrality	Degree centrality
GAPDH	360.406	0.011	68
EGFR	431.981	0.011	67
AKT1	329.569	0.011	66
BCL2	261.498	0.011	65
TNF	335.329	0.010	63
CASP3	156.416	0.010	58
SRC	266.413	0.009	54
ESR1	124.018	0.009	53
MMP9	111.792	0.009	50
PTGS2	283.483	0.009	45

Degree Centrality reflects a node's importance based on its direct connections. Betweenness Centrality is the number of shortest paths through a node in a network. Closeness Centrality is used to measure node importance.
